# Bioaerosol sampling for airborne bacteria in a small animal veterinary teaching hospital

**DOI:** 10.3402/iee.v3i0.20376

**Published:** 2013-08-06

**Authors:** Tisha A. M. Harper, Shelley Bridgewater, Latoya Brown, Patricia Pow-Brown, Alva Stewart-Johnson, Abiodun A. Adesiyun

**Affiliations:** 1 College of Veterinary Medicine, University of Illinois, Urbana, IL, USA; 2 Faculty of Medical Sciences, School of Veterinary Medicine, The University of the West Indies, St. Augustine, Trinidad, West Indies

**Keywords:** veterinary hospital, airborne, bioaerosol sampling, bacteria

## Abstract

**Background:**

Airborne microorganisms within the hospital environment can potentially cause infection in susceptible patients. The objectives of this study were to identify, quantify, and determine the nosocomial potential of common airborne microorganisms present within a small animal teaching hospital.

**Methods:**

Bioaerosol sampling was done initially in all 11 rooms and, subsequently, weekly samples were taken from selected rooms over a 9-week period. Samples were collected twice (morning and afternoon) at each site on each sampling day. The rooms were divided into two groups: Group 1, in which morning sampling was post**-**cleaning and afternoon sampling was during activity, and Group 2, in which morning sampling was pre-cleaning and afternoon sampling was post-cleaning. The total aerobic bacterial plate counts per m^3^ and bacterial identification were done using standard microbiological methods.

**Results:**

A total of 14 bacterial genera were isolated with the most frequent being *Micrococcus* spp. followed by species of *Corynebacterium*, *Bacillus*, and *Staphylococcus*. There was a significant interaction between location and time for rooms in Group 1 (*p*=0.0028) but not in Group 2 (*p*>0.05). Microbial counts for rooms in Group 2 were significantly greater in the mornings than in the afternoon (*p*=0.0049). The microbial counts were also significantly different between some rooms (*p*=0.0333).

**Conclusion:**

The detection of significantly higher airborne microbial loads in different rooms at different times of the day suggests that the probability of acquiring nosocomial infections is higher at these times and locations.

Bacteria have been documented to be responsible for nosocomial infections which are of both public health and economic importance and are increasingly becoming a concern among veterinary patients and practitioners. Nosocomial infections are a common cause of morbidity for affected animals leading to a prolonged hospital stay as well as increased use of resources to treat such infections (1, 2). They are also of particular concern in surgical patients and the development of surgical site infections (1). It has also been reported that some nosocomial infections are zoonoses (3).

Microorganisms such as *Klebsiella* spp., *Salmonella* spp., *Serratia marcescens*, *Clostridium difficile*, and *Acinetobacter baumannii* have been identified as causative organisms of nosocomial infections in hospitalized dogs and cats (4–8). Methicillin-resistant *Staphylococcus aureus* (MRSA) is now one of the organisms of major concern in both large and small animal hospitalized patients (9–12).

Although causative organisms have been isolated from patients (13, 14), hospital personnel and inanimate objects or surfaces (15) in the environment (10), the role of airborne bacteria as a source of infection or their role in environmental transmission of nosocomial infections is unclear (10, 16). In veterinary surgery, contamination of surgical suctions tips with bacteria in room air may pose an important risk to the development of surgical site infections (17). Prevention of infections requires the identification and control of the potential sources of microbial contamination (18).

Bioaerosol monitoring in hospitals can provide information for epidemiological investigation of nosocomial infectious diseases, research into airborne microorganisms’ spread and control as well as provide information regarding the current status of operations (19). One of the recommended goals for surveillance programs for nosocomial infections in veterinary hospitals is to estimate the rate of pathogen shedding and evaluate environmental contamination with infectious agents (20).

The objectives of this study were to determine the prevalence, types, and quantity of airborne microorganisms in a small animal clinic at a veterinary teaching hospital; and to assess variations in microbial counts at different times and locations within the teaching hospital. Our working hypothesis was that there would be no difference in microbial counts in rooms sampled pre- and post-cleaning. An additional hypothesis was that there would be no difference in microbial counts between rooms.

## Materials and methods

### Rooms sampled

Eleven rooms were sampled in the small animal clinic. The rooms selected were those in which animals were housed or through which there was a regular flow of animals on a daily basis. The rooms sampled were the large operating room (LOR), small operating room (SOR), large examination room (LER), small examination room (SER), special procedures room (SP), surgery preparation room (SPR), radiology room (RAD), cat room (CR), recovery room (RR), kennels room (KR), and isolation room (IR). Bioaerosol samples were taken once weekly over a 9-week period. Morning (AM) and afternoon (PM) samplings were done on each sampling day, which was the same day each week. In week 1, all locations were sampled once. The rooms were subsequently divided into two groups. Group 1 (LER, LOR, RAD, SER, SOR, SP, SPR) consisted of rooms where procedures were performed whereas Group 2 were the rooms where patients were housed. The rooms were sampled from weeks 2 to 9 using the protocol shown in Table 1. For Group 1, AM sampling was post-cleaning, samples were taken after the rooms were cleaned for the day and afternoon (PM) sampling was during routine activity. For Group 2, AM samplings were pre-cleaning, samples were taken before daily cleaning of the rooms were performed and PM samplings were post-cleaning, that is during activity. The order of room sampling was randomized on each sampling day, however, due to biosecurity reasons, the IR was always sampled last, following which a 1:10 chlorine bleach solution was used to decontaminate the wheels and legs of the sampling trolley and stand.

**Table 1:** Sampling schedule for the study

		Sampling week								
		
Room^a^	Group	1	2	3	4	5	6	7	8	9
SOR	1	•	•		•		•		•	
RAD	1	•	•		•		•		•	
CR	2	•	•		•		•		•	
KR	2	•	•		•		•		•	
IR	2	•	•		•		•		•	
LER	1	•		•		•		•		•
SER	1	•		•		•		•		•
SP	1	•		•		•		•		•
SPR	1	•		•		•		•		•
LOR	1	•		•		•		•		•
RR	2	•		•		•		•		•

• Sampling areas.

a SOR = small operating room, RAD = radiology room, CR = cat room, KR = Kennel room, IR = isolation room, LER = large examination room, SER = small examination room, SP = special procedures room, SPR = surgery preparation room, LOR = large operating room, RR = recovery room.

### Sample collection

An SKC^®^ Standard Biostage Impactor (SKC Biostage Cat No. 225-9611) was used for bioaerosol sampling. The impactor was mounted on a 5 ft tall stand and connected to a sampling pump, model S37MYHCB-1452 (GAST Manufacturing Inc., Motor Division, St. Louis, MO, USA) with flexible tubing. The pump was set to the manufacturer's flow specifications of 28.3 L/min. The impactor allowed for air to be drawn directly onto blood agar (BA) plates. Sampling times of 2 and 4 min were used for each room. These time periods were selected to ensure that total aerobic plate counts (TAPC) per m^3^ were obtained for at least one sampling time without overgrowth on the plate, that is a dilution method. Room doors were closed and no movement into or out of rooms was allowed during the sampling times. The impactor was steam sterilized before each sampling day. It was sterilized with 99% ethanol between rooms. A total of 319 samples were collected and immediately taken to the laboratory for further processing.

### Sample processing

The BA plates were incubated aerobically (Thermo Fisher Scientific Inc., MA, USA) at 37°C overnight. The TAPCs were enumerated using a Quebec Dark field colony counter (Cambridge Instruments Inc., NY, USA) and the morphology of the colonies documented. Representative colonies of each type of growths on each plate were then individually inoculated into 0.5 mL of brain heart infusion (BHI) broth in cryovials. The cryovials were mixed using a vortex mixer and incubated at 37°C overnight after which 0.5 mL of 50% glycerol was added to the contents of the cryovials, mixed thoroughly on a vortex mixer and stored at −20°C until further testing.

## Isolation of aerobic bacteria

Cultures stored at −20°C were thawed at room temperature and used to inoculate BA plates which were incubated overnight at 37°C. Pure colonies were stained by Gram's method to determine the Gram-positive and Gram-negative aerobic bacteria. All Gram-negative bacteria were inoculated onto MacConkey agar and incubated at 37°C overnight.

### Identification of microbial organisms

All Gram-positive and Gram-negative aerobic bacteria were identified using exhaustive biochemical tests and serological tests (21, 22).

### Statistical analysis

Rooms in the hospital were divided into two groups. One group comprised seven rooms and the other four rooms. For each group of rooms a series of contingency tables were generated to examine the frequencies of genus isolation. These tables included an overall genus frequency table with frequencies summed over room, week, and time of day, followed by two-way tables to assess if the location (room), time of day, or week had an effect on the frequency of genus isolation over the 9-week period. Associations between location, time of day, and week, and frequency of microbe isolation were assessed using chi-square tests (where required, an exact or a Monte Carlo estimate of the exact test was used). Presence or absence of the most frequent genus (*Micrococcus*) was also modeled using Generalized Estimating Equations (GEE) to simultaneously assess the effects of location, time of day, and week. Significance was set at alpha = 0.05. All analyses were performed using SAS (version 9.1.3 Service Pack 4, Cary, NC, USA).

## Results

### Frequency of isolation of bacteria

*Group 1:* A total of 13 genera were isolated namely *Enterobacter aerogenes*, species of *Acinetobacter*, *Aerococcus*, *Alcaligenes*, *Bacillus*, *Citrobacter*, *Corynebacterium*, *Klebsiella*, *Kurthia*, *Micrococcus*, *Pseudomonas*, *Serratia*, and *Staphylococcus*. The four most frequently isolated genera were *Micrococcus* (36.6%), *Corynebacterium* (16.8%), *Bacillus* (16.0%), and *Staphylococcus* (14.5%), and the differences in frequency of distribution of genera of bacteria were statistically significant (*p*<0.0001) (Table 2). Using GEE modeling, the frequencies of Group 1 isolates did not significantly differ with time of day sampled (*p*>0.05). The most frequent genus present in both AM and PM sampling was *Micrococcus*, with frequencies of 19.1% and 17.6% respectively (Table 2). The frequency of distribution of *Micrococcus* was found to be significantly higher (*p*<0.05) than found for other bacteria either in AM or PM sampled. *Alcaligenes* and *Klebsiella* were not detected during AM sampling but present during PM sampling. *Citrobacter*, *Pseudomonas*, and *Serratia* were present during AM sampling but were not detected during PM sampling (Table 2).

**Table 2:** Frequency distribution of aerobic bacteria in Group 1 rooms

	Time of sampling		
	
	AM	PM	Total
	
Organisms	Frequency (%)* of distribution of bacteria		
*Enterobacter aerogenes*	3 (2.3)	1 (0.8)	4 (3.1)
*Acinetobacter* spp.	2 (1.5)	4 (3.1)	6 (4.6)
*Aerococcus* spp.	1 (0.8)	0 (0.0)	1 (0.8)
*Alcaligenes* spp.	0 (0.0)	2 (1.5)	2 (1.5)
*Bacillus* spp.	15 (11.5)	6 (4.6)	21 (16.1)
*Citrobacter* spp.	1 (0.8)	0 (0.0)	1 (0.8)
*Corynebacterium* spp.	11 (8.4)	11 (8.4)	22 (16.8)
*Klebsiella* spp.	0 (0.0)	2 (1.5)	2 (1.5)
*Kurthia* spp.	1 (0.8)	1 (0.8)	2 (1.6)
*Micrococcus* spp.	25 (19.1)	23 (17.6)	48 (36.7)
*Pseudomonas* spp.	1 (0.8)	0 (0.0)	1 (0.8)
*Serratia* spp.	2 (1.5)	0 (0.0)	2 (1.5)
*Staphylococcus* spp.	14 (10.7)	5 (3.8)	19 (14.5)

AM = morning, PM = afternoon.

* Based on number of times a particular bacterium was isolated/total number of times (131) all bacteria were isolated.

*Group 2:* A total of 11 genera were isolated comprising *E. aerogenes*, *E. coli*, *Acinetobacter*, *Alcaligenes*, *Bacillus*, *Citrobacter*, *Corynebacterium*, *Klebsiella*, *Micrococcus*, *Serratia*, and *Staphylococcus*. The four genera that were highly distributed were *Micrococcus* (34.9%), *Staphylococcus* (16.9%), *Corynebacterium* (15.7%), and *Bacillus* (15.7%) as shown in Table 3. Using GEE modeling, the frequencies of Group 2 isolates did not significantly differ with time of day (*p*>0.05). The most frequent genus present for both AM and PM sampling was *Micrococcus*, with frequencies of 18.1% and 16.9%, respectively (Table 3). The frequency of isolation of *Micrococcus* was found to be significantly higher than detected for other bacteria (*p*<0.05). *Citrobacter* was not detected during AM sampling but present during PM sampling. *E. aerogenes* and *Serratia* spp. were present during AM sampling but were not detected during PM sampling (Table 3).

**Table 3:** Frequency distribution of aerobic bacteria in Group 2 rooms

	Time of sampling		
	
	AM	PM	Total
	
Organism	Frequency (%)* of distribution of bacteria		
*Enterobacter aerogenes*	1 (1.2)	0 (0.0)	1 (1.2)
*Escherichia coli*	3 (3.6)	1 (1.2)	4 (4.8)
*Acinetobacter* spp.	2 (2.4)	1 (1.2)	3 (3.6)
*Alcaligenes* spp.	1 (1.2)	1 (1.2)	2 (2.4)
*Bacillus* spp.	9 (10.8)	4 (4.8)	13 (15.6)
*Citrobacter* spp.	0 (0.0)	1 (1.2)	1 (1.2)
*Corynebacterium* spp.	8 (9.6)	5 (6.0)	13 (15.6)
*Klebsiella* spp.	1 (1.2)	1 (1.2)	2 (2.4)
*Micrococcus* spp.	15 (18.1)	14 (16.9)	29 (35.0)
*Serratia* spp.	1 (1.2)	0 (0.0)	1 (1.2)
*Staphylococcus spp*.	9 (10.8)	5 (6.0)	14 (16.8)

AM = morning; PM = afternoon.

* Based on number of times a particular bacterium was isolated/total number of times (83) all bacteria were isolated.

### Time and location of sampling

**Fig. 1:**
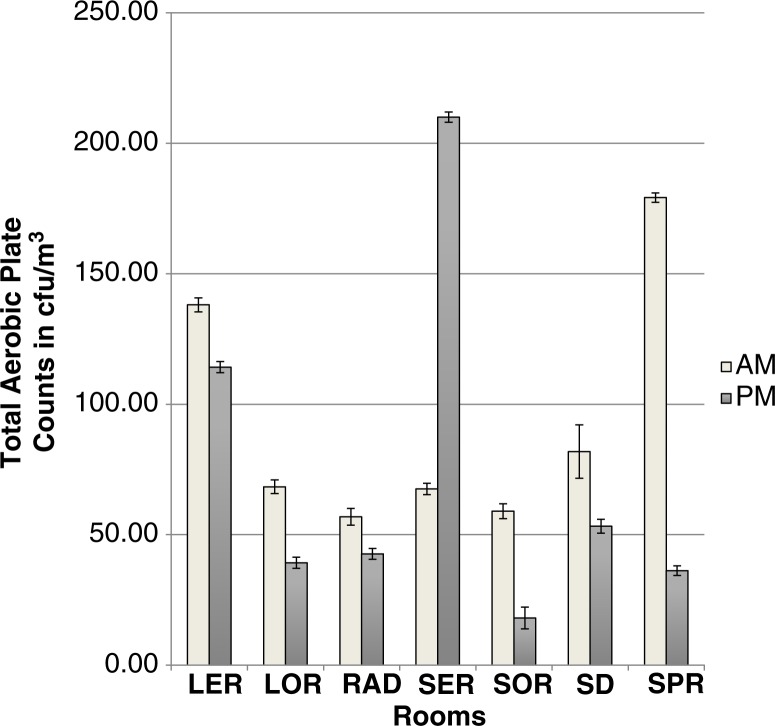
Geometric mean±SD of total air plate counts of Group 1 rooms (*p*=0.0028).

*Group 1:* Using the geometric mean/m^3^, the mean microbial count for AM sampling for Group 1 was 81.3±3.1 cfu/m^3^ and 57.1±3.2 cfu/m^3^ for PM sampling but the differences were not statistically significant (*p*>0.05). There was a significant interaction between location and time for rooms in Group 1 (*p*=0.0028). A comparison of the microbial load of air samples of the rooms revealed significant differences (*p*=0.0287) in the AM samples but not in the AM samples (*p*=0.4767). Furthermore, for each of SER, SOR, and SPR, mornings and afternoons had significantly different microbial counts (*p*<0.05). Counts in SER were significantly higher during the PM sampling (Fig. 1). Microbial counts in the mornings and afternoons did not differ significantly for each of the remaining rooms in Group 1.

**Fig. 2:**
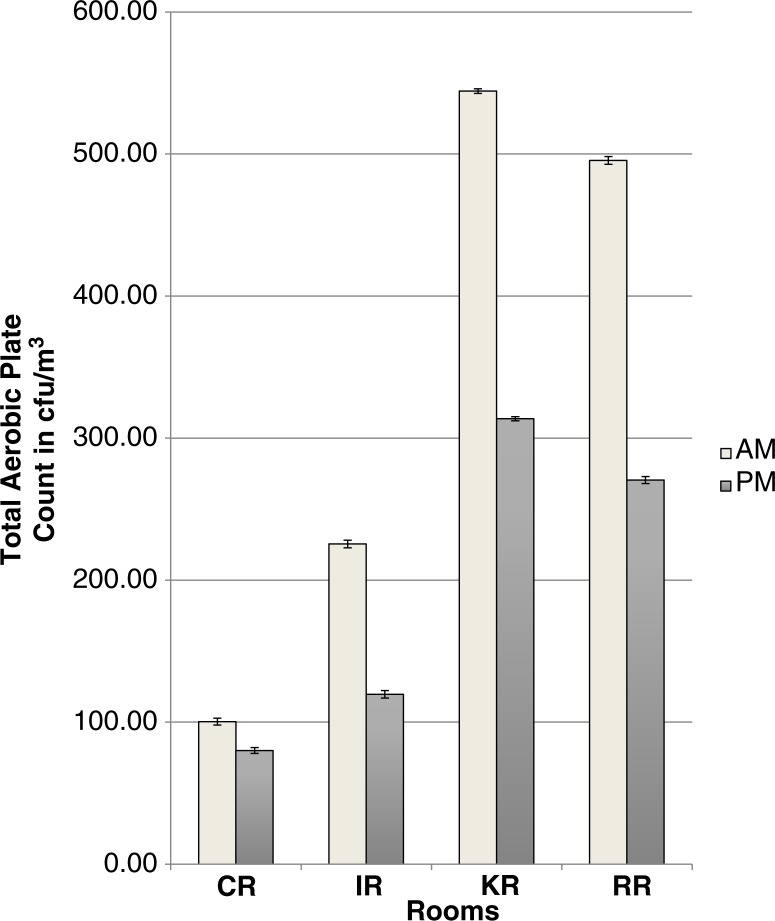
Geometric mean±SD of total air plate counts of Group 2 rooms.

*Group 2:* Using the geometric mean, the mean microbial count for AM sampling was 291.3±3.0 cfu/m^3^ and for PM sampling was 172.9±2.5 cfu/m^3^ and the difference was statistically significant (*p*=0.0049). There was also a significant difference in microbial counts between the different rooms (*p*=0.0333). No interaction between time and location was observed in Group 2 (*p*>0.05); however, the Tukey–Kramer procedure for multiple testing showed that only rooms CR and KR had significantly different microbial counts (*p*=0.045) from each other (Fig. 2). In Group 2, rooms RR and KR were found to have higher AM counts compared to PM counts and this difference was significant (*p*<0.05).

A comparison of the geometric means of the bacterial loads of both Groups 1 and 2 rooms showed statistically significantly higher values for Group 2 rooms in both AM and PM samples.

### Week of sampling

There was significant difference between week of sampling and microbial levels in Groups 1 and 2 (*p*<0.05).

## Discussion

Some of the bacterial genera isolated in this study have been previously implicated in veterinary nosocomial infections, specifically *Klebsiella*, *Serratia*, *Acinetobacter*, *Staphylococcus*, and *Pseudomonas* (4–6, 23, 24). Airborne contamination with bacterial organisms implicated in nosocomial infections has been described in human hospitals (25, 26).

*Micrococcus*, the most frequent genus isolated from both Group 1 and Group 2 rooms in this study has not, to our knowledge, been reported to cause nosocomial infections in dogs and cats. It has mainly been implicated in skin infections in these species (27). A possible reason for its high frequency of isolation may be due to the fact that the organism is a common skin inhabitant and thus is easily shed into the environment on desquamated epithelial skin cells. The possibility that *Micrococcus* spp. may cause nosocomial infections can, however, not be completely ignored considering its high frequency of detection in this study.

*Staphylococcus* was the second most frequently isolated genus from both groups. It has been suggested that the environment may be an important source of MRSA infection, which is becoming of major concern in veterinary hospitals (10, 14, 23). Airborne transmission of MRSA seems important in the acquisition of nasal carriage, and/or spread of the organism on skin scales that are liberated into the air during daily activities in human hospitals, for example bed making (28).

Group 2 consisted of the four rooms in which patients are housed. It was therefore not a surprise that *E. coli* was present during both AM and PM sampling. PM sampling represented post-cleaning; however, patients were always present in the rooms thus the presence of *E. coli* was not unexpected. The frequency of isolation of *E. coli* was less during PM sampling, that is post-cleaning. It is important, however, to note that *E. coli* was not isolated from any of the Group 1 rooms which included the operating, examination and treatment rooms.

Overall, microbial counts in Group 2 rooms were significantly higher than in Group 1 rooms. This was not surprising as Group 2 rooms housed patients throughout the day and night. Movement of patients and personnel in and out of kennels during the day most likely accounted for the higher counts resulting from the dispersal of organisms in the air. In a study on the potential for airborne transmission of *Clostridium difficile* in a human hospital, they also found there was circulation and dispersal of organisms during movement, particularly personnel movement, opening and closing of doors, disturbance of already contaminated inanimate objects and lack of regular cleaning of air vents (25).

Microbial counts in Group 1 rooms did not differ significantly between the rooms in the mornings. This finding could be explained, in part, by the fact that morning sampling in Group 1 was done immediately post-cleaning, therefore, this suggests consistency in the cleaning regimen in these rooms in the mornings. The significant differences between Group 1 rooms during PM sampling is a reflection of the activity in the rooms during the day. SER is a room with very little ventilation which may have led to the higher PM counts. The finding that SOR and SPR had higher AM counts compared to their PM counts was unexpected as sampling occurred after cleaning of the operating room. This finding may be explained in part, by the fact that SPR is a three-walled room with one side open to a corridor, thereby readily exposed to the inflow of contaminated air, as earlier described by others (29).

Overall, Group 2 rooms had a significantly higher microbial load in the morning than in the afternoon. This correlates with the pattern of cleaning in the rooms. AM sampling was pre-cleaning and so higher counts would be expected compared with post-cleaning PM counts. There were significant differences between the counts in the different rooms in Group 2. Although the study design did not include a record of patient numbers at the time of sampling, the differences may have been related to the patient numbers in the different rooms. In Group 2 rooms, the RR and KR had significantly higher AM counts when compared to their PM counts. This was cause for concern, particularly for the RR as post-surgical patients recover in this area.

There are no documented standards for the evaluation of microbiological air quality in human hospitals (30) or veterinary hospitals. There are clean-room classifications established by the European Union and the United States (31); however, there is no consensus on the density of airborne microflora at which the risk of infection may be increased. Recognition of the risk of airborne dissemination provides an opportunity to reduce transmission. Airborne biological material can be investigated by collecting air samples and measuring viable microorganisms (30).

The microbial load in an environment will vary from day to day and at different times of the day (16). We designed our study to capture what would be representative bacteria during or following major events in the clinic, for example pre- and post-cleaning and including major activity times of the day. Some authors suggest that short sampling times only represent a snapshot of the microbes present; however, the disadvantage of longer sampling times is overgrowth of plates (32). Our hope was that repeated sampling over a 9-week period would give us a fair representation of the organisms present. Another limitation of the study was that sampling was carried out with the impactor at a height of 5 ft and this may not necessarily have represented the true bacterial flora in each room. Published studies suggest that sampling at lower heights equivalent to the working level of the rooms be used (32). A third limitation of the study is that air sampling was performed in two groups of rooms with different functions and the systemic sampling in relation to cleaning procedures in the morning and evenings in these two groups of rooms. This therefore makes it difficult to separate the effect of room function and sampling relative to time of cleaning in the analysis.

## Conclusions

It was concluded that there was a significant difference in air microbial loads in rooms sampled pre- and post-cleaning, with a general decrease in the microbial counts post-cleaning. It was also concluded that there were significant differences in microbial counts between rooms with higher counts in rooms that housed patients compared to rooms that did not. *Micrococcus* spp. were determined to be most frequently detected in the air of all rooms sampled with a potential to cause nocosomial infections in the hospital studied, although the significance is unknown. Air exchange and ventilation, and cleaning protocols throughout the hospital, particularly in the SOR, need to be re-evaluated.

## References

[1] Brown DC, Conzemius MG, Shofer F, Swann H (1997). Epidemiologic evaluation of postoperative wound infections in dogs and cats. J Am Vet Med Assoc.

[2] Dunning D, Slatter D (2003). Surgical wound infection and the use of antimicrobials. Textbook of small animal surgery.

[3] Morley PS (2002). Biosecurity of veterinary practices. Vet Clin North Am Food Anim Pract.

[4] Fox JG, Beaucage CM, Folta CA, Thornton GW (1981). Nosocomial transmission of *Serratia marcescens* in a veterinary hospital due to contamination by benzalkonium chloride. J Clin Microbiol.

[5] Francey T, Gaschen F, Nicolet J, Burnens AP (2000). The role of *Acinetobacter baumannii* as a nosocomial pathogen for dogs and cats in an intensive care unit. J Vet Intern Med.

[6] Glickman LT (1981). Veterinary nosocomial (hospital-acquired) Klebsiella infections. J Am Vet Med Assoc.

[7] Uhaa IJ, Hird DW, Hirsh DC, Jang SS (1988). Case-control study of risk factors associated with nosocomial *Salmonella krefeld* infection in dogs. Am J Vet Res.

[8] Weese JS, Armstrong J (2003). Outbreak of *Clostridium difficile*-associated disease in a small animal veterinary teaching hospital. J Vet Intern Med.

[9] Tomlin J, Pead MJ, Lloyd DH, Howell S, Hartmann F, Jackson HA (1999). Methicillin-resistant *Staphylococcus aureus* infections in 11 dogs. Vet Rec.

[10] Weese JS, DaCosta T, Button L, Goth K, Ethier M, Boehnke K (2004). Isolation of methicillin-resistant *Staphylococcus aureus* from the environment in a veterinary teaching hospital. J Vet Intern Med.

[11] Weese JS, Dick H, Willey BM, McGeer A, Kreiswirth BN, Innis B (2006). Suspected transmission of methicillin-resistant *Staphylococcus aureus* between domestic pets and humans in veterinary clinics and in the household. Vet Microbiol.

[12] Weese JS, Rousseau J, Willey BM, Archambault M, McGeer A, Low DE (2006). Methicillin-resistant *Staphylococcus aureus* in horses at a veterinary teaching hospital: frequency, characterization, and association with clinical disease. J Vet Intern Med.

[13] Boerlin P, Eugster S, Gaschen F, Straub R, Schawalder P (2001). Transmission of opportunistic pathogens in a veterinary teaching hospital. Vet Microbiol.

[14] Hanselman BA, Kruth S, Weese JS (2008). Methicillin-resistant staphylococcal colonization in dogs entering a veterinary teaching hospital. Vet Microbiol.

[15] Wright JG, Tengelsen LA, Smith KE, Bender JB, Frank RK, Grendon JH (2005). Multidrug-resistant *Salmonella typhimurium* in four animal facilities. Emerg Infect Dis.

[16] Dharan S, Pittet D (2002). Environmental controls in operating theatres. J Hosp Infect.

[17] Sturgeon C, Lamport AI, Lloyd DH, Muir P (2000). Bacterial contamination of suction tips used during surgical procedures performed on dogs and cats. Am J Vet Res.

[18] Stocks GW, Self SD, Thompson B, Adame XA, O'Connor DP (2010). Predicting bacterial populations based on airborne particulates: a study performed in nonlaminar flow operating rooms during joint arthroplasty surgery. Am J Infect Contr.

[19] Li CS, Hou PA (2003). Bioaerosol characteristics in hospital clean rooms. Sci Total Environ.

[20] Morley PS (2004). Surveillance for nosocomial infections in veterinary hospitals. Vet Clin North Am Equine Pract.

[21] Macfadin JF (2000). Biochemical tests for identification of medical bacteria.

[22] Baer EF, Gray RJH, Orth DS (1976). Compendium of methods for the microbial examination of foods.

[23] Duquette RA, Nuttall TJ (2004). Methicillin-resistant Staphylococcus aureus in dogs and cats: an emerging problem?. J Small Anim Pract.

[24] Greene CE, Dearmin MG, Greene CE (2006). Surgical and traumatic wound infections. Infectious diseases of the dog and cat.

[25] Best EL, Fawley WN, Parnell P, Wilcox MH (2012). The potential for airborne dispersal of *Clostridium difficile* from symptomatic patients. Clin Infect Dis.

[26] Kumari DN, Haji TC, Keer V, Hawkey PM, Duncanson V, Flower E (1998). Ventilation grilles as a potential source of methicillin-resistant *Staphylococcus aureus* causing an outbreak in an orthopaedic ward at a district general hospital. J Hosp Infect.

[27] Ihrke PJ, Greene CE (2006). Integumentary infections. Infectious diseases of the dog and cat.

[28] Solberg CO (2000). Spread of *Staphylococcus aureus* in hospitals: causes and prevention. Scand J Infect Dis.

[29] Greene VW, Velsey D, Bond RG, Michaelson GS (1961). Microbiological contamination of hospital air II. Quantitative studies. Appl Microbiol.

[30] Ortiz G, Yague G, Segovia M, Catalan V (2009). A study of air microbe levels in different areas of a hospital. Curr Microbiol.

[31] Gangneux JP, Robert-Gangneux F, Gicquel G, Tanquerel JJ, Chevrier S, Poisson M (2008). Bacterial and fungal counts in hospital air: comparative yields for 4 sieve impactor air samplers with 2 culture media. Infect Contr Hosp Epidemiol.

[32] Kung'u J (2004). Limitations and considerations in air sampling, sample analysis and result interpretation for airborne mould spores. Inoculum.

